# Tobacco Next?

**Published:** 1920-03-20

**Authors:** 


### TOBACCO NEXT ?

History tell us that, since 1586 when Raleigh made himself at the same time both distinguished and notorious by growing tobacco on his estate and deliberately smoking in public, a persistent and fluctuating opposition to the followers of this habit has raged. Not always, one is glad to reflect, was that vindictive intensity displayed with which certain Popes excommunicated smokers one and all, or Christian IV. of Denmark had all smokers publicly whipped, or the Chinese dispensed their famous punishments by maiming. At times, however, we may read beheadings and even wholesale slaughters were carried out upon perpetrators of the smoking crime. Yet, in spite of such powerful foes, the custom has steadily increased until for two or more centuries it has been practically universal. This history stands, indeed, in marked contrast with that of the much condemned consumption of alcohol, but the comparison though nowadays perhaps amusing, when one remembers the latter's ancient popularity, serves but little useful purpose in considering the latest craze, namely, the prohibition of tobacco as well as alcohol.

Let us admit that smoking, learned from the New World savage, has, and always will have, many who, with certain American writers, denounce it as a barbarous custom. But let us at the same time remember there are smokers, and smokers. A famous contributor to these pages would have portrayed them to a line in his "Human Temperaments." Might he now stand by us to bring shrieking to light the absurdities of this insidious negative policy which tends to grow and grow!

At least, if the new movement is real and seriously intended let us, before attempting for ever to banish tobacco, take due consideration of what mysterious social and economic conditions must lie behind a demand so enormous and widely established. On general lines, if the use of tobacco is to be classed as a dissipation, at least it must be admitted as a dissipation in a very mild form, and probably the individual robbed of his right to smoke is perfectly justified in claiming a corresponding suppression of the tea and coffee of those responsible for the legislation. The medical critic may, on a strictly scientific basis, disagree, but, nevertheless, there is more than a little similarity between these two "vices." Each may do harm. Irritable heart, heightened blood-pressure, and tobacco ambyopia are perfectly genuine conditions. So are the well-known effects of tannin and caffeine. And yet considering the enormous number of people daily subjected to such "poisonings" is it not amazing, if the evil be really so great, that, in actual practice, relatively few whose woes can truthfully be ascribed to such causes ever enter our surgeries? No doubt many would, in a medical sense, be better off without these things, but in life as it is to-day there are other considerations. For the mass of the people, they have come to occupy an all-important place among the few antidotes to an ever-present mental strain.

There is, of course, no question that excess leads to harm, but we venture the opinion that under average conditions it is the abuse of tobacco, rather than its usually accepted use, which lies at the root of most of its evil effects. After all, abuse of most things is either obnoxious or in some way damaging.

Let us, therefore, before we talk of prohibition, and before our New World cousins think to legislate, first see how we can reduce the tension under which we live, reduce the fierceness of a growing struggle for existence, and solve some of the essential problems presented by massed humanity and overcrowded living. Let us increase by all possible measures the means for natural relaxation, and we shall have gone far towards finding a substitute, not only for smoking but for many more health damaging habits.

